# Dynamic Neuromuscular Stabilization Exercise and Chronic Lumbar Disc Herniation: Effects on Pain, Mobility, and Trunk Endurance—A Randomized Controlled Trial

**DOI:** 10.1002/hsr2.71611

**Published:** 2025-12-14

**Authors:** Bahare Ameri, Ali Fatahi, Raheleh Nasr Abadi, Rozhin Molavian

**Affiliations:** ^1^ Department of Physical Education and Sport Sciences, CT.C. Islamic Azad University Tehran Iran

**Keywords:** functional disability, lumbar disc herniation, muscular endurance, pain intensity, range of motion

## Abstract

**Background:**

This randomized controlled trial investigated the effects of an 8‐week Dynamic Neuromuscular Stabilization (DNS) exercise program on pain intensity, lumbar range of motion, functional disability, and trunk muscle endurance in women with chronic lumbar disc herniation.

**Methods:**

A total of 30 women aged 30–40 years with chronic lumbar disc herniation (L4–L5 or L5–S1, pain duration ≥ 3 months, Visual Analog Scale (VAS) 3–7) were randomly assigned to either an experimental group (*n* = 15), performing DNS exercises (45–60 min, 3 times/week for 8 weeks), or a control group (*n* = 15), continuing routine physical activity (self‐directed walking/light stretching). Pain intensity was measured using the VAS, lumbar range of motion using the modified Schober test, functional disability using the Oswestry Disability Index (ODI), and trunk muscle endurance using the McGill endurance test. Data were analyzed using Paired and independent *t*‐tests to assess within‐ and between‐group differences (SPSS v25, *p* < 0.05).

**Results:**

The control group showed no significant changes (*p* > 0.05). In contrast, the experimental group demonstrated significant improvements in all outcome variables (*p* < 0.05). Postintervention comparisons revealed significant between‐group differences favoring the experimental group across all measures (*p* < 0.007). Pain intensity decreased by 4.0 (95% CI: −4.5 to −3.5), lumbar range of motion increased by 5.0 cm (95% CI: 4.2 to 5.8), functional disability decreased by 26.2 (95% CI: −29.1 to −23.3), and trunk flexor endurance increased by 24.4 s (95% CI: 20.1 to 28.7).

**Conclusion:**

An 8‐week DNS program significantly reduced pain, improved lumbar mobility, decreased functional disability, and enhanced trunk muscle endurance in women with chronic lumbar disc herniation, suggesting its potential as an effective rehabilitation strategy.

## 1 Introduction

Low back pain (LBP) is a highly prevalent musculoskeletal disorder that significantly impacts a large segment of the population, contributing to diminished quality of life and increased disability, particularly among women [1, 2, 3]. One of the primary causes of LBP is lumbar disc herniation, often triggered by mechanical stressors, including bending and compressive forces affecting the lumbar spine. Chronic LBP, characterized by pain persisting for more 3 months, is a multifaceted condition that leads to functional impairments and imposes considerable economic and social burdens [4, 5].

In recent decades, there has been a significant advancement in understanding the interplay between muscular stability and the effective functioning of the movement system. Research indicates that dysfunction in the muscles responsible for spinal stabilization can adversely affect movement control, which may hinder the ability to maintain neutral joint positioning, contributing to segmental instability in the lower back, ultimately resulting in pain [6]. Effective spinal stabilization, involving coordinated muscle, ligament, and nervous system function, is critical for maintaining spinal integrity [7].

To address associated complications related to disk herniation conditions, nonpharmacological methods, including the *dynamic neuromuscular stability technique*, have been developed. These techniques aim to mitigate the adverse effects of such conditions without relying on medication. Research indicates that conservative management strategies, such as physical therapy and specific exercise regimens, can effectively reduce symptoms and improve patient outcomes. Furthermore, the implementation of these noninvasive approaches not only enhances recovery but also contributes to cost savings in healthcare by potentially decreasing the need for surgical interventions [6].

One of the latest sports rehabilitation techniques is the dynamic neuromuscular stability technique, which, in addition to strengthening the muscular system, also involves the nervous system [7].

Dynamic Neuromuscular Stabilization (DNS) is a *manual and rehabilitative approach* designed to optimize the motor system, grounded in the scientific principles of *developmental kinesiology*. This technique emphasizes the importance of core stability and the coordinated function of the diaphragm, which plays a crucial role in both postural control and movement efficiency [8, 9]. DNS focuses on activating the intrinsic stabilizers of the spine and ensuring proper breathing patterns before engaging in functional movements, thereby enhancing overall motor performance and rehabilitation outcomes [7, 10]. The approach is particularly beneficial in treating various neurological and musculoskeletal conditions like lumbar disc herniation, as it integrates the principles of *kinetic chain dynamics* and regional interdependence of the body's systems [11, 12]. By employing DNS, practitioners aim to restore optimal movement patterns and improve functional stability, which is essential for both athletic performance and rehabilitation [7]. The basic techniques of DNS therapeutic principles include general training of central stability, practice of the body pattern in favor of and against the movement of the limbs to step forward and support, training of evolutionary postural movement models to stabilize the position, paying attention to the stability of each part from the chains involved in the muscles, correspondence of the postural function with the force of the movement phase, stabilization training and breathing pattern, keeping the spine stable, progression of movements from easy to advanced, not using pathological movement patterns and starting sports activities after the patient's awareness by the doctor [13, 14].

So far, various studies have investigated the importance of using DNS exercises. The use of these exercises in the elderly population [15], multiple sclerosis [11], people with postural abnormalities of scoliosis [16], head forward [17], chronic nonspecific back pain patients [18], chronic neck pain [19], history of concussion [19], and so forth. Recently, Frank et al. highlighted DNS's effectiveness in reducing pain and improving function in chronic LBP [20].

With the goals of affecting the factors of balance and trunk function, walking function, improving physical posture, improving respiratory condition, on the amount of pain, range of motion and selected muscle endurance of the trunk, motor function, the quality of life has been discussed, and the results of evidence studies emphasize the importance and effect of the practice principles of DNS on the mentioned variables. Recently, in the studies of Najafi Ghagholestani et al. [21], Ghavipanje et al. [22], Mousavi and Mirsafaei Rizi [23], Karati et al. [24], Venkatsan et al. [25], Lim et al. [18], and Alvani et al. [26], investigating the effects of using DNS exercises on the severity and disability of chronic back pain. The results of the mentioned studies have emphasized the effectiveness of DNS exercises on reducing pain intensity and improving the functional ability of people with chronic back pain.

Given the importance of modern rehabilitation techniques for chronic back pain caused by lumbar disc herniation, this study focuses on the effects of DNS exercises on functional and physical factors in women with chronic back pain. To the researcher's knowledge, this is the first study to specifically investigate the impact of DNS exercises in women with chronic back pain due to lumbar disc herniation. The study aims to answer whether an 8‐week DNS exercise program can influence pain intensity, range of motion, functional disability, and muscular endurance of the trunk in this population. The findings may provide valuable insights into the potential benefits of DNS for improving functional and physical outcomes in women suffering from chronic back pain caused by lumbar disc herniation.

## 2 Materials and Methods

The research conducted was an applied‐interventional study with a pretest–posttest design and a control group. G*power software with significance level (*α*) set to 0.05 and statistical power of 80%, was used to determine that a minimum of 26 subjects were needed, and 30 subjects were selected from women with lumbar disc herniation, considering potential dropouts. The criteria for entering the research include: having mental health, having a history of pain caused by chronic lumbar disc herniation for at least 3 months, suffering from a lumbar disc between the L4–L5 and L5–S1 vertebrae, not being pregnant, having chronic back pain in the range of 3–7 Visual analog MIAS; and criteria for exiting the project include: occurrence of pain at rest and during activity, difficulty in performing the exercise protocol and inability to perform exercise movements, noncooperation and withdrawal during the exercise protocol, lack of regular attendance in two continuous sessions and three in an intermittent training session was considered.

The researcher tried to identify the subjects by referring to the medical center and hospital of Tehran city as well as virtual pages. People with a history of lumbar disc herniation confirmed by a doctor or test, as well as having a history of at least 3 months of nonradiating back pain and chronic back pain in the range between 3 and 7 on the Visual Analog Scale (VAS). Among these, 30 women with chronic lumbar disc herniation with an age range of 30–40 years located in Tehran were randomly divided into two groups (one training group and one control group). The training group performed 45–60 min three sessions per week for 8 weeks, and the control group did their routine sports activities. After the subjects were divided before the pretest stage, the subjects completed an individual informed consent form. Then, the individual information form such as name and surname, age, height, weight, and questions about medical history was provided to those interested in participating in the research.

The tools needed in this research include personal information form, personal consent form, medical history form and physical activity readiness questionnaire (PAR‐Q), VAS, modified Schuber test, Oswestry test, and McGill test.

Pre‐ and posttests were conducted identically for both groups, assessing the following outcomes:

- Pain intensity: measured using the VAS (0–10 cm, reliability: 0.88) [27].
- Lumbar range of motion: assessed via the modified Schober test (validity: *R* = 0.90, reliability: 0.90–0.96) [28, 29].
- Functional disability: evaluated with the Oswestry Disability Index (ODI, reliability: 0.84) [30].
- Trunk muscle endurance: measured using the McGill endurance test, comprising four timed tests (trunk flexor, extensor, right and left lateral flexor endurance), assessing the ability to hold standardized positions (e.g., plank for flexor endurance, prone extension for extensor endurance) [23].

The validity and scientific reliability of VAS have been confirmed in numerous studies abroad, and in Iran, the reliability of this scale has been confirmed with a correlation coefficient of 0.88 [27], also the Schuber test has high validity and reliability [28]. The validity of this test has been highly correlated with radiography (*R* = 0.90); also, intra‐examiner reliability of 0.90 and inter‐examiner reliability of 0.96 have been reported for this test [29].

In earlier studies, the validity and reliability of the Oswestry disability questionnaire in assessing the level of back pain and disability in daily activities have been established, with its reliability reported at 84% [30].

**Figure 1:**
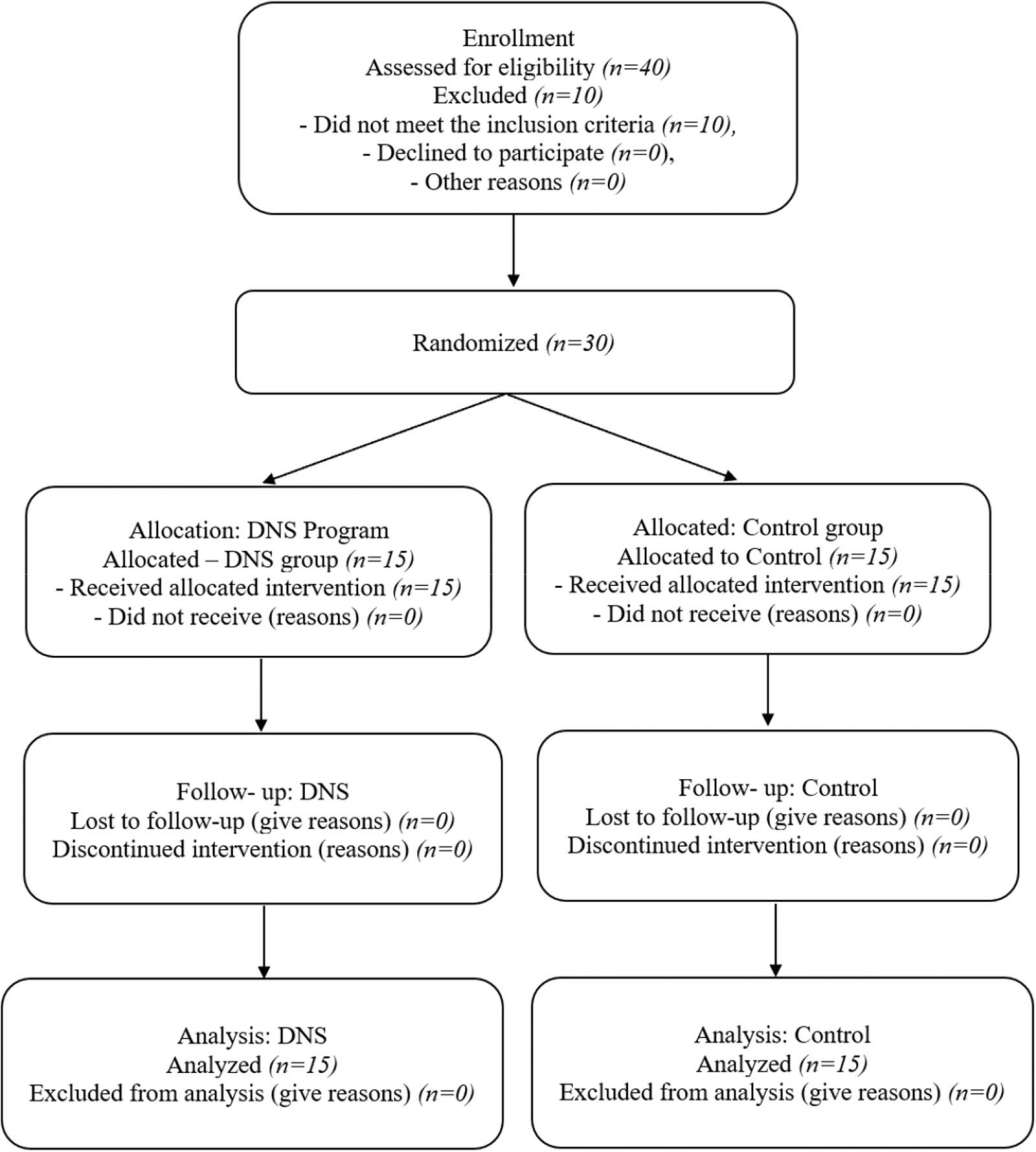
A CONSORT flow diagram.

Participants were randomized using a computer‐generated random number sequence, with allocation concealed via sealed opaque envelopes prepared and implemented by an independent researcher to ensure impartiality. Participants were assigned to an experimental group (*n* = 15) or a control group (*n* = 15). Outcome assessors were blinded to group allocation, though blinding of participants and therapists was not feasible due to the intervention's nature. All participants provided written informed consent and completed a demographic and medical history questionnaire. A CONSORT flow diagram (Figure 1) illustrates participant recruitment, allocation, follow‐up, and analysis.

### 2.1 Intervention

The experimental group performed a supervised 8‐week DNS program (3 sessions/week, 45–60 min), based on Kolar's principles [13]. Exercises progressed from simple (e.g., diaphragmatic breathing, lying movements) to advanced (e.g., squatting, kneeling with resistance), as detailed in Appendix A. Progression was controlled based on correct execution and previous session intensity [21]. The control group continued routine physical activity, defined as self‐directed walking or light stretching (30–45 min, 3 times/week), without structured intervention.

#### 2.1.1 Implementation of the Training Program

The research training program is based on Pavel Kolar's theory and consists of three sections: simple, medium, and advanced activities. The DNS exercises were designed with three levels: simple, medium, and advanced, reflecting muscle chains. In the first sessions, the exercises were performed with a lower intensity and in the final sessions they increased the intensity [21]. The warm‐up phase involved walking, running, and stretching. The DNS group's exercises include breathing, lying down, rolling over, sitting, kneeling, squatting, and standing up. Each exercise builds on the previous level and higher levels are not allowed until mastery of the lower level. Overload and gradual increase of each exercise was controlled and specified based on correct implementation and pressure from the previous session [21].

#### 2.1.2 Posttest Implementation

After the DNS training protocol was implemented for 8 weeks, the posttest phase was implemented in the subjects of both training and control groups. To evaluate the dependent variables of the research, at this stage, exactly the same tests as the pretest were done. After summarizing the research steps and obtaining information from the samples, the research data will be summarized and entered into SPSS software.

The current research was approved by the Ethics Committee of the Research Institute of Physical Education and Sports Sciences and had the code of ethics SSRI.REC‐2304‐2166.

### 2.2 Statistical Analysis

Descriptive statistics (means, standard deviations) and inferential statistics (paired and independent *t*‐tests) were used. Normality was confirmed using the Shapiro–Wilk test, and homogeneity of variance was verified with Levene's test. To address the risk of Type I error due to multiple comparisons, a Bonferroni correction was applied (*α* = 0.05/7 = 0.007 for seven outcome variables). Effect sizes (Cohen's *d*) and 95% confidence intervals were calculated for significant outcomes. All analyses were prespecified and conducted using SPSS v25 [31, 32].

## 3 Results

### 3.1 Participant Flow

Figure 1 shows the CONSORT flow diagram. Of 40 screened participants, 30 met the inclusion criteria and were randomized (15/group). All participants completed the study (30/30, 100% retention).

### 3.2 Baseline Characteristics

Table 1 shows the demographic characteristics of the subjects by group. No significant between‐group differences were found (*p* > 0.05).

**Table 1:** Demographic characteristics of the subjects (independent *t*‐test, *p* < 0.05).

Variable	Group (*N* = 15)	Mean	SD	*t*	Sig	ES
Age (years)	Training	34.06	2.81	−1.241	0.225	0.1
	Control	35.40	3.06			
Weight (kg)	Training	66.46	5.66	0.342	0.735	0.12
	Control	65.73	6.07			
Height (cm)	Training	156.06	4.31	0.440	0.664	0.15
	Control	164.33	4.80			
BMI (kg/m^2^)	Training	23.40	1.95	0.668	0.510	0.09
	Control	22.93	1.86			
The amount of pain (ND)^a^	Training	6.33	1.11	0.156	0.877	0.087
	Control	6.26	1.22			
History of pain (year)	Training	9.20	1.93	0.154	0.879	0.14
	Control	9.06	2.73			

a No dimension.

Table 2 shows the results of the paired and independent *t*‐test for the pretest–posttest comparison of research variables in two training and control groups. Based on these results, in the experimental group, there was a significant difference between the average scores obtained between the pretest and the posttest in the variables of back pain intensity (*p* = 0.001; *t* = 7.338), range of motion of the back (*p* = 0.001; *t* = −4.459), back functional disability (*p* = 0.001; *t* = 11.856), trunk flexor muscle endurance (*p* = 0.001; *t* = 9.607), extensor muscle endurance (*p* = 0.001; *t* = 7.784), endurance of right lateral flexor muscles (*p* = 0.001; *t* = 6.915), and endurance of left lateral flexor muscles (*p* = 0.001; *t* = 5.829).

**Table 2:** Paired and independent *t*‐test results (*p* < 0.05, Bonferroni‐corrected).

Variable	Group	Pretest(Mean ± SD)	Posttest(Mean ± SD)	Paired *t*‐test (df = 14)	*p*	ES	Independent *t*‐test (df = 28)	*p*	ES
Trunk flexor muscle endurance	Training	26.5 ± 2.16	50.9 ± 6.42	−9.60	0.001*	0.926	8.510	0.001**	0.93
	Control	25.6 ± 3.15	26.5 ± 6.45	−0.19	0.851	0.044			
Trunk extensor muscle endurance	Training	20.3 ± 4.31	34.5 ± 3.69	−7.78	0.001*	0.682	7.426	0.001**	0.68
	Control	21.3 ± 6.93	21.3 ± 4.74	0.32	0.755	0.021			
Endurance of right lateral flexor muscles	Training	26.4 ± 3.74	22.33 ± 3.49	−6.91	0.001*	0.639	7.067	0.001**	0.64
	Control	16.2 ± 7.35	17.13 ± 4.10	−1.60	0.132	0.052			
Endurance of left lateral flexor muscles	Training	22.3 ± 3.49	22.33 ± 3.49	−5.83	0.001*	0.475	6.283	0.001**	0.48
	Control	16.4 ± 6.07	17.13 ± 4.10	0.94	0.364	0.015			
Pain intensity	Training	6.1 ± 0.16	2.1 ± 0.16	7.34	0.001*	0.549	−9.043	0.001**	0.55
	Control	6.1 ± 0.11	6.1 ± 0.22	−1.33	0.204	0.038			
Functional disability	Training	48.46 ± 6.13	22.3 ± 4.52	11.856	0.001*	0.536	−11.595	0.001**	0.54
	Control	47.4 ± 3.48	47.7 ± 2.62	−0.274	0.788	0.002			

* Significant differences between pre–post in control and training groups.

** Significant differences between posttest of control and training groups.

Also, the results of the independent *t*‐test show that a significant difference between the results of the two training and control groups in the mean posttest scores in the variables of back pain intensity (*p* = 0.001), range of motion of the back (*p* = 0.010), functional disability of the back (*p* = 0.001), endurance of trunk flexor muscles (*p* = 0.001), endurance of extensor muscles (*p* = 0.001), endurance of right lateral flexor muscles (*p* = 0.001) and left (*p* = 0.001).

However, effect sizes indicated high effectiveness for all exercises, with trunk flexor muscle endurance exercises demonstrating the highest effectiveness (ES = 0.926).

## 4 Discussion

Due to the continuous human effort to find the most efficient method in the shortest time, this particular research seeks to discover a treatment method that has not yet been investigated for its effectiveness in removing lumbar disc herniation. This randomized controlled trial demonstrates that an 8‐week DNS program significantly improves pain intensity, lumbar range of motion, functional disability, and trunk muscle endurance in women with chronic lumbar disc herniation, consistent with prior studies [5, 21, 22, 23, 24, 25, 26].

The results of the recent research showed that there was a significant difference between the pretest and the posttest in the back pain intensity variable in the exercise group; however, there was no significant difference between the mean intensity of back pain in the control group between the pre‐ and posttest stages (*p* < 0.05). Also, there was a significant difference between the two training and control groups in the mean posttest scores of the back pain intensity variable (*p* = 0.001) (3.766 decrease in the training group). The results of the recent research showed that there was a significant difference between the pretest and the posttest in the back pain intensity variable in the exercise group; however, there was no significant difference between the mean intensity of back pain in the control group between the pre‐ and posttest stages (*p* < 0.05). Also, there was a significant difference between the two training and control groups in the mean posttest scores of the back pain intensity variable (*p* = 0.001) (3.766 decrease in the training group). By restoring the coordination, flexibility, endurance, and strength of the muscles through appropriate exercises, the therapeutic exercise will bring back the balance and proper functioning of the muscles and joints, and in this way, the problem of disc herniation will be basically solved DNS likely reduces pain by improving coordination and strength of deep spinal stabilizers, reducing stress on the lumbar spine [33, 34] and also the results of the current research on the effect of DNS exercises on reducing pain in people with chronic back pain have been consistent with previous studies. The experimental group's improvements align with Najafi Ghagholestani and colleagues, Venkatesan and colleagues, and Frank et al., who reported DNS‐related reductions in pain and disability in chronic LBP [20, 21, 25]. The previous studies demonstrate that altering the movement pattern and utilization of the deep back muscles, which are crucial for stabilizing this region, can lead to pain and muscle imbalance in individuals with chronic back pain. Consequently, it may have a detrimental impact on the performance of such individuals [35]. Therefore, the positive effects of these exercises on pain intensity and performance of these patients can be justified for this reason. DNS's focus on core stability and motor control may mitigate pain by reducing compensatory movements. In this regard, Inani and Selkar reported a more significant effect of central exercises on reducing pain and improving the performance of patients with chronic back pain compared to traditional exercises, presenting core‐focused exercises as more effective than traditional exercises for pain and function [36].

Frank and colleagues noted that DNS enhances spinal stabilizer activation, potentially reducing muscle atrophy and pain sensitivity [7]. Increased lumbar range of motion (5.0 cm, 95% CI: 4.2–5.8) in the experimental group aligns with Mousavi and Mirsafaei Rizi, who reported improved flexibility with DNS [23]. However, the main difference between the present research and the previous studies is the type of subjects. The findings of the present study have supported the effect of DNS exercises on improving the range of motion of women with chronic lumbar disc herniation. As mentioned, DNS exercises develop and control sensory‐motor trunk muscles and central muscles of the body, and with the development of trunk stability, additional forces damaging the spine are reduced that reduces pain. These factors will be secondarily effective in increasing the range of motion of the trunk area. The weakness of the back muscles and the increase in pain in these patients cause limitation and decrease the range of motion of the back. Enhanced trunk stability likely reduces compensatory movements, improving mobility.

Functional disability decreased significantly (26.2, 95% CI: −29.1 to −23.3), consistent with Karati and colleagues, Venkatesan and colleagues, and others, reflecting improved motor control and daily function [24, 25].

The results of intragroup differences showed that in the endurance variables of trunk flexor muscles (increase of 11.833 s), extensor muscle endurance (increase of 6.067 s), endurance of right lateral flexor muscles (increase of 4.933 s), and endurance of left lateral flexor muscles (increase of 3.167 s). There was a significant difference between pretest and posttest in the training group (*p* < 0.05). However, there was no significant difference between the pretest and posttest stages in the control group in the average of the muscular endurance variables of the trunk region (*p* < 0.05). Also, there is a significant difference between the two training and control groups in the mean posttest scores of the endurance variables of trunk flexor muscles (increase of 23.933 s in the training group), endurance of extensor muscles (increase of 13.266 s in the training group), endurance of the right lateral flexor muscles (there was an increase of 9.66 s in the training group), and the endurance of the left lateral flexor muscles (an increase of 7.60 s in the training group) (*p* < 0.05). The results of the current research on the effect of DNS exercises on increasing muscular endurance of the trunk in people with chronic back pain are in line with the studies of Mousavi and Mirsafaei Rizi. Trunk muscle endurance improvements (e.g., flexor: 24.4 s, 95% CI: 20.1–28.7, ES = 0.93) support Mousavi and Mirsafaei Rizi, with the high effect size indicating DNS's efficacy in strengthening core muscles [23]. The findings of the present study support the effect of DNS exercises on increasing the muscular endurance of the trunk of women with chronic lumbar disc herniation. Various studies have been conducted to increase the muscular endurance of the trunk through various stability exercises such as central stability exercises, pilates, back stabilizers, and yoga in people with lumbar disc herniation, and the results of the present study were consistent with them. Mousavi and Mirsafaei Rizi studied the effect of central stability and DNS exercises on pain, flexibility, balance, muscle endurance, and quality of life of men with nonspecific chronic back pain [23]. The results showed that there was a significant increase in trunk muscle endurance in the DNS training group [23]. In line with these studies, Mousavi and Fatahi and Taşpınar and colleagues supported the effect of Pilates exercises on reducing disability and increasing the strength of central muscles, trunk flexibility (normal standing and trunk extension), and endurance of central muscles (left side and right side). Other stability exercises, such as Pilates and yoga, have shown similar benefits [37, 38].

Reduced trunk muscle strength increases spinal stress, contributing to pain. DNS likely strengthens multifidus and other extensor muscles, enhancing endurance [39, 40]. People who have less strength and endurance in trunk muscles are more affected by structural pressures. This issue may cause inappropriate pressure on the spine and cause back pain in people with lumbar disc herniation [39]. In people with back pain, it is important to pay attention to the strengthening of the muscle groups that open the spine [40]. Therefore, according to the mentioned research, it seems that the exercise protocol used in this research was probably able to improve and increase the size of the multifidus muscles and other extensor muscles and finally increase their strength.

The findings of the present study support the effect of DNS exercises on increasing the level of movement ability of women with chronic lumbar disc herniation. In addition to back pain in people with chronic lumbar disc herniation, reduced functional disability is another common symptom of these patients, which affects their daily activities [41]. Karati and colleagues have considered the effects of the DNS approach on clinical results, including functional balance and quality of life in elderly patients with nonspecific chronic back pain, as a positive case [24]. Venkatsan and colleagues, comparing yoga and DNS training in chronic back pain, have confirmed an increase in the level of functional ability through the ODI following DNS training [25]. Alvani and colleagues showed that 8 weeks of DNS training improves functional disability and dynamic balance in athletes with nonspecific chronic back pain, and it has been suggested that DNS exercises are effective exercises in the rehabilitation program of athletes with nonspecific chronic back pain [26]. Additionally, past studies have emphasized the role of stabilizing exercises, such as Pilates [37, 38], back stabilizers [42], and the McKenzie method [43], as well as central stability [44], in reducing the functional disability of people with chronic lumbar disc herniation. Improved functional ability aligns with Karati and colleagues and Alvani and colleagues, who reported enhanced balance and function with DNS [24, 26].

It seems that the nature of DNS exercises used in this research with the aim of increasing endurance, muscle strength and reducing pain intensity, increasing the level of functional motor ability in the trunk area, increasing the muscle strength of the lower limbs, and also creating sufficient stability in the spine area on increasing the muscular endurance time of the various tests in the present research was effective in women suffering from chronic lumbar disc herniation. DNS's focus on endurance, strength, and stability likely drives these improvements.

### 4.1 Limitations

The study's small sample size (*n* = 30), short intervention duration (8 weeks), lack of long‐term follow‐up, and inclusion of only women limit generalizability. The absence of participant and therapist blinding may introduce bias, though assessor blinding mitigates this risk.

### 4.2 Future Directions

Larger trials with diverse populations, longer interventions, and follow‐up periods are needed to confirm findings. Comparative studies with interventions like Pilates or yoga could clarify DNS's relative efficacy.

## 5 Conclusion

An 8‐week DNS program significantly reduces pain, enhances lumbar mobility, decreases functional disability, and improves trunk muscle endurance in women with chronic lumbar disc herniation. These findings support DNS as a promising rehabilitation strategy, warranting further research to optimize its application.

## Author Contributions

Conceptualization: Bahare Ameri and Ali Fatahi. Methodology: Bahare Ameri and Ali Fatahi. Software: Ali Fatahi and Raheleh Nasr Abadi. Data curation: Ali Fatahi. Investigation: Ali Fatahi and Bahare Ameri. Validation: Ali Fatahi. Formal analysis: Ali Fatahi and Raheleh Nasr Abadi. Supervision: Ali Fatahi and Rozhin Molavian. Visualization: Ali Fatahi. Project administration: Ali Fatahi and Raheleh Nasr Abadi. Writing – original draft: Ali Fatahi and Rozhin Molavian. Writing – review and editing: Ali Fatahi and Rozhin Molavian.

## Disclosure

The lead author Ali Fatahi affirms that this manuscript is an honest, accurate, and transparent account of the study being reported; that no important aspects of the study have been omitted; and that any discrepancies from the study as planned (and, if relevant, registered) have been explained.

## Ethics Statement

The current research was approved by the Ethics Committee of the Research Institute of Physical Education and Sports Sciences and had the code of ethics SSRI.REC‐2304‐2166.

## Consent

Informed consent was obtained from all subjects involved in the study.

## Conflicts of Interest

The authors declare no conflicts of interest.

## Data Availability

The data presented in this study are available on request from the corresponding author.

## 1

**DNS Training Levels**

**Different levels of breathing exercises**

**Table:**

Level	Breath exercise
A1	Teaching and practicing diaphragmatic breathing while resting (lying, sitting, standing)
A2	Practice maintaining diaphragmatic breathing while statically holding basic DNS positions
A3	A2 + various movements of one hand or one leg
A4	A2 + various combined movements of an arm and leg (positive or negative) in a single movement plane
A5	A2 + various combined movements of one arm and leg (for or against) in two different movement planes
A6	Diaphragmatic breathing exercise while performing DNS basic movements

**Different levels lying on the back 90–90**

**Table:**

Level	Leg position	Movement lying on the back
B1	Lean against the wall	Keep moving and focus on diaphragmatic breathing
B2		B1 + flexion and extension of one hand
B3		B1 + simultaneous flexion and extension of both hands in the elbow (B3‐) and arm (B3‐2) and combined (B3‐3) joints
B4		B1 + simultaneous movement of two hands in the elbow and arm joints in different motion planes with and without dumbbells
B5		B1 + simultaneous flexion and extension of one hand and one leg
B6	Without leaning on the wall	B4 no dumbbells
B7		B4 use dumbbells
B8		B5
B9		Simultaneous movement of one arm and one leg in two different planes
B10		Ball pressure between both thighs and hip extension/flexion movement
B11		Ball pressure between both thighs with single/pair movement of hands in different planes of motion
B12		B11 + extension/flexion of the knees
B13		B12 + use of dumbbells or theraband
B14		B11 + extension/flexion of knees and hip joints
B15		Reverse B11 (using ring theraband instead of ball)
B16		B15 + hip extension
B17		B15 + use dumbbells
B18		B15 + using a circular band around the legs and using dumbbells
B19		B18 + hip extension
B20		Combining movement with movement is wrong
B21		B20 + use dumbbells

*Note:* Keeping the trunk steady in these movements is a kind of nonconscious exercise of the blind muscles. From movement B2 onwards, breathing is done unconsciously, in the form of a diaphragm. In complex movements, it should be noted that the knees do not assume a varus/valgus position.

**Different levels lying on the stomach**

**Table:**

Level	Move lying on the stomach
C1	Keep moving and focus on diaphragmatic breathing
C2	Bilateral pressure on the ground
C3	C2 + weight transfer to the flexed leg
C4	C3 + lifting the trunk
C5	C1 + horizontal flexion of the arms
C6	C5 + straight leg hyperextension
C7	C5 + dumbbell
C8	C6 + we keep the straight leg bent and the dumbbell behind the bent knee
C9	Pressing both hands on the ground and simultaneously separating the trunk and thighs from the ground, and the weight falling on the knees and palms

*Note:* Basic position: hyperextension movement of the neck and upper body, putting weight on the forearm, and flexion of the hip and knee joints in one leg. It should be noted that when lifting the trunk, there should be no flexion or hyperextension in the back.

**Different levels of motion to roll**

**Table:**

Level	Action to roll
D1	Rolling to the right and left sides with a short range with diaphragmatic exhalation and back from rolling with diaphragmatic exhalation.
D2	Stabilization in short‐amplitude side roll (with three diaphragmatic breaths)
D3	D2 + (with the time of 5 diaphragmatic breaths)
D4	D2 + flexion and extension of the hand
D5	D2 + movement of the opposite arm and leg in different motion planes
D6	D4 + dumbbell
D7	D5 + dumbbells (hands and back of knees)
D8	D1 + dumbbells (two hands and two knees behind)
D9	D1 + ball pressure between both hands + circular theraband stretching around both thighs
D10	D2 + a small tap on the ball between both hands + stretching the band around both thighs
D11	D2 + a small tap on the ball between the hands/thighs
D12	D1 + with long range
D13	D3 + with long range
D14	D13 + movement of the opposite arm and leg in different motion planes
D15	D12 + dumbbells (two hands and two knees behind)
D16	D12 + ball pressure between both hands + circular theraband stretching around both thighs and around both legs
D17	D13 + a small tap on the ball between both hands + stretching the band around both thighs
D18	D13 + small strike on the ball between both hands + hip extension
D19	Combining the movement of rolling with the movement of sitting from the side
D20	D19 + dumbbell

*Note:* Keeping the trunk steady in these movements is a kind of nonconscious exercise of the blind muscles. In complex movements, it should be noted that the knees do not assume a varus/valgus position.

**Different levels of side sitting movement**

**Table:**

Level	Side sitting motion
E1	Keeping the movement static and focusing on diaphragmatic breathing
E2	Hand movement in different movement planes
E3	Leg movement in different movement planes
E4	Simultaneous movement of arms and legs in different motion planes
E5	E2 + dumbbell
E6	E4 + theraband
E7	Lifting the trunk from the ground with the pressure of the lower hand
E8	E7 + dumbbell
E9	E8 + arm and leg movement in different movement planes
E10	Combination of side sitting movement with oblique sitting movement
E11	E10 + dumbbell

*Note:* Keeping the trunk steady in these movements is a kind of nonconscious exercise of the blind muscles.

**Different levels of inclined sitting motion**

**Table:**

Level	Inclined sitting motion
F1	Keeping the movement static and focusing on diaphragmatic breathing
F2	Hand movement in different movement planes
F3	Leg movement in different movement planes + flexion and extension of knee and thigh joints
F4	Simultaneous movement of arms and legs in different motion planes
F5	F2 + dumbbells and theraband
F6	F4 + dumbbells and theraband
F7	Lifting the pelvis off the ground
F8	F7 + simultaneous movement of arms and legs in different motion planes
F9	F8 + dumbbells and theraband
F10	Combination of inclined sitting with kneeling movement
F11	F10 + dumbbells

*Note:* Keeping the trunk steady in these movements is a kind of nonconscious exercise of the blind muscles.

**Different levels of tripod movement**

**Table:**

Level	Tripod movement
G1	Keeping the movement static and focusing on diaphragmatic breathing
G2	Movement of each hand in different planes of motion (bent knee)
G3	The movement of each leg (from the knee and thigh joints) in different movement planes (bent knee)
G4	Simultaneous movement of the opposite arm and leg in different planes of movement (bent knee)
G5	G2 (straight knee)
G6	G3 (straight knee)
G7	G4 (straight knee)
G8	Staying steady in the three‐legged movement (with the time of two diaphragmatic breaths)
G9	G5 + theraband stretching around both thighs
G10	G9 + hand dumbbell
G11	G6 + theraband stretching around both thighs
G12	G7 + theraband stretching around both thighs and around both legs
G13	Staying steady in the three‐legged movement (with the time of five diaphragmatic breaths)

*Note:* Keeping the trunk steady in these movements is a kind of nonconscious exercise of the blind muscles

**Different levels of kneeling motion**

**Table:**

Level	Kneeling motion
H1	Keeping the movement static and focusing on diaphragmatic breathing
H2	Single and simultaneous movement of hands in different movement planes
H3	H2 + dumbbell
H4	H2 + theraband
H5	Knee extension with short range
H6	H5 + movement of the hands in the sagittal plane
H7	H5 + movement of hands in different movement planes
H8	H7 + dumbbell
H9	Extension of the knees with full range simultaneously with the movement of the hands in the sagittal plane
H10	Combining kneeling movements with squat movements
H11	H10 + dumbbell

*Note:* Keeping the trunk steady in these movements is a kind of nonconscious exercise of the blind muscles.

**Different levels of kneeling motion**

**Table:**

Level	Squat movement
K1	Doing squats with a short range and focusing on diaphragmatic breathing (both hands in front of the chest in the horizontal plane)
K2	Keeping the movement static and focusing on diaphragmatic breathing (both hands in front of the chest in the horizontal plane)
K3	K1 + (two hands next to the ears on the frontal screen)
K4	K3 + with long range + dumbbells
K5	K2 + with long range + movement of hands in different movement planes
K6	K5 + dumbbells and threbands
K7	K6 + wrist plantar flexion
K8	K1 + with long range + movement of the hands in different planes of the rack with dumbbells and theraband
K9	K8 + circular band around the thighs

*Note:* Keeping the trunk steady in these movements is a kind of nonconscious exercise of the blind muscles.

**DNS training protocol used in this research (experimental group in 8 weeks)**

**Table:**

Session	First 10 min	Second 10 min	Third 10 min	Fourth 10 min
1	A1–B1–B2	C1–C2–G1	E1–E2–F1–F2	H1–H2–K1–K2
2	A1–B2–B3	C1–C2–C3–G1	E1–E3–F3	H1–H2–K1–K2
3	A1–B3–B4	C2–C3–G1–G2	E2–E3–F2–F3	H2–H3–K2–K3
4	A1–B4–B5	C1–C4–G1–G2–G3	E4–F4	H2–H3–H4–K3–K4
5	A1–B6–D1	C5–G4	E2–E4–E5–F2–F4–F5	H1–H5–K4–K5
6	A1–B6–B7–D1–D2	C5–C6–G5	E4–E6–F4–F6	H5–H6–K5–K6
7	A1–B7–B8–D1–D2	C6–C7–G5–G6	E4–E5–E6–F4–F5–F6	H5–H6–K5–K6
8	A1–B9–D1–D3	C8–G5–G6–G7	E6–E7–F6–F7	H5–H6–K6
9	A1–B10–D3–D4	C4–C8–G7–G8	E7–E8–F7–F8	H7–K6–K7
10	A1–B10–B11–D5–D6–D7	C6–C7–G8–G9	E6–E7–E8–F6–F7–F8	H7–H8–K7
11	A1–B11–B12–D8‐D9	C6–C7–G9–G10	E8–E9–F8–F9	H7–H8–K7–K8
12	A1–B12–B13–D10–D11	C7–C8–G9–G10	E7–E8–E9–F7–F8–F9	H7–H8–H9–K8
13	A1–B14–D10–D11–D12	C8–C9–G10–G11	E7–E10–F10–H1	H9–K8–K9
14	A1–B15–D13–D14	C7–C8–G10–G11	E7–E11–F11–H1	H9–H10–K9
15	A1–B16–B17–D15–D16	C6–C7‐G11‐G12	E11‐F11‐H3	E10‐F10‐H10
16	A1–B18–B19–D17–D18	C6–C7–G13	E11–F11–H7	E10–F10–H10
17	B20–D19	C9–G11–G12–G13	E11–F11–H8	CGU
18	B21–D20	C9–G11–G12–G13	E11–F11–H9	CGU
